# Titin fragment is a sensitive biomarker in Duchenne muscular dystrophy model mice carrying full-length human dystrophin gene on human artificial chromosome

**DOI:** 10.1038/s41598-025-85369-5

**Published:** 2025-01-13

**Authors:** Yosuke Hiramuki, Miwa Hosokawa, Kayo Osawa, Taku Shirakawa, Yasuhiro Watanabe, Ritsuko Hanajima, Hiroyuki Kugoh, Hiroyuki Awano, Masafumi Matsuo, Yasuhiro Kazuki

**Affiliations:** 1 Department of Chromosome Biomedical Engineering, School of Life Science, Faculty of Medicine, Tottori University, 86 Nishi-cho, Yonago, Tottori, 683‑8503 Japan; 2 Department of Chromosome Biomedical Engineering, Graduate School of Medical Science, Tottori University, 86 Nishi-cho, Yonago, Tottori, 683‑8503 Japan; 3 Faculty of Health Sciences, Kobe Tokiwa University, 2-6-2 Otani-cho, Nagata, Kobe, 653-0838 Japan; 4 Division of Neurology, Department of Brain and Neurosciences, Faculty of Medicine, Tottori University, 86 Nishi-cho, Yonago, Tottori, 683‑8503 Japan; 5 Research Initiative Center, Organization for Research Initiative and Promotion, Tottori University, 86 Nishi-cho, Yonago, 683-8503 Japan; 6 Graduate School of Science and Technology and Innovation, Kobe University, 1-1 Rokkodai-cho, Nada, Kobe, 657-8501 Japan; 7 Chromosome Engineering Research Group, The Exploratory Research Center on Life and Living Systems (ExCELLS), National Institutes of Natural Sciences, 5-1 Higashiyama, Myodaiji, 444-8787 Okazaki, Aichi Japan

**Keywords:** Duchenne muscular dystrophy, Dystrophin, Creatine kinase, Titin, Human artificial chromosome, Biomarkers, Diseases

## Abstract

Duchenne muscular dystrophy (DMD) is an X-linked recessive disorder caused by mutations of the dystrophin gene, which spans 2.4 Mb on the X chromosome. Creatine kinase (CK) activity in blood and titin fragment levels in urine have been identified as biomarkers in DMD to monitor disease progression and evaluate therapeutic intervention. However, the difference in the sensitivity of these biomarkers in DMD remains unclear. Previously, we generated transchromosomic mice carrying the full-length human dystrophin gene on a human artificial chromosome (DYS-HAC1) vector. The human dystrophin derived from DYS-HAC1 improved pathological phenotypes observed in *DMD-null* mice, which lack the entire 2.4 Mb of the dystrophin gene. In this study, we compared the values of plasma CK activity and urine/plasma titin fragment levels in wild-type (WT), DYS-HAC1, *DMD-null*, and DYS-HAC1; *DMD-null* mice. Plasma CK activity and urine/plasma titin fragment levels in *DMD-null* mice were significantly higher than those in WT mice. Although plasma CK activity showed no significant difference between WT and DYS-HAC1; *DMD-null* mice, urine/plasma titin fragment levels in DYS-HAC1; *DMD-null* mice were higher than those in WT mice. Human dystrophin in DYS-HAC1;* DMD-null* mice drastically improved muscular dystrophy phenotypes seen in *DMD-null* mice; however, the proportion of myofibers with central nuclei in DYS-HAC1; *DMD-null* mice had a tendency to be slightly higher than that in WT mice. These results suggest that urine/plasma titin fragment levels could be a more sensitive biomarker than plasma CK activity.

**Supplementary Information:**

The online version contains supplementary material available at 10.1038/s41598-025-85369-5.

## Introduction

Duchenne muscular dystrophy (DMD) is an X-linked recessive muscle disorder characterized by severe muscle wasting and is the most common muscular dystrophy, affecting 4.6 in 100,000 people worldwide^1,2^. DMD is caused by mutations of the dystrophin gene, which spans 2.4 Mb on the X chromosome. Dystrophin protein contains N-terminus, rod, cysteine, and C-terminus domains, and functions as a link between the cytoskeleton and the basal lamina to stabilize muscle structure. Mutations of dystrophin cause dysfunction of this linkage in skeletal muscles and heart. Many laboratories have developed DMD animal models carrying mutations of the dystrophin gene, ranging from invertebrate to large mammalian models, to deepen our understanding of the molecular mechanisms involved in this disorder and to develop biomarkers and therapies for it^3^. For example, male *DMD-null* mice, which lack the entire 2.4 Mb of the dystrophin gene, display severe muscular dystrophy characterized by degenerating myofibers with concomitant cellular infiltration and regenerating myofibers with centrally located nuclei^4^.

Biomarkers such as proteins, metabolites, and microRNAs for monitoring the disease progression and evaluating therapeutic effects have been developed in DMD patients and animal models^5–15^. Plasma/serum creatine kinase (CK) activity is widely used as a biomarker for muscle damage and muscular dystrophies including DMD. CK is an enzyme that reversibly catalyzes the reaction of creatine and ATP to phosphocreatine and ADP and is released from muscle cells into the blood upon muscle injury. As a noninvasive biomarker, urine titin fragment levels have been used in DMD patients and animal model^16–21^ and its fragments are also detected in patients with Becker muscular dystrophy, Limb-girdle muscular dystrophy, Fukuyama congenital muscular dystrophy, and myotonic dystrophy type 1 but not in neurogenic spinal muscular atrophy^16,22,23^. Titin is one of the largest genes in humans, consisting of 364 exons, and functions as a molecular blueprint, a molecular spring, and a mediator of mechanical signaling in muscle cells^24^. Titin is a substrate of some proteases, such as matrix metalloproteinase-2, and the titin fragments produced by such digestion are released from muscle cells into the blood or urine^25^.

Human/mouse artificial chromosome vectors are created by deleting endogenous genes on a chromosome and are capable of carrying megabase-size genomic regions with replication/segregation as an independent chromosome^26^. Using mouse artificial chromosome, Down syndrome model mice carrying the long arm of human chromosome 21^27^, human antibody-producing mice carrying human immunoglobulin loci^28^, and human drug-metabolizing enzyme-producing rats carrying UGT2 cluster and CYP3A cluster^29^ have been developed. In addition to the mouse artificial chromosome, we previously generated DYS-HAC1 mice carrying the 2.4 Mb human dystrophin gene on a human artificial chromosome^30,31^. DYS-HAC1 mice express both mouse and human dystrophin in skeletal muscles and heart, while male DYS-HAC1; *DMD-null* mice expressing only human dystrophin were generated by crossing male DYS-HAC1 with female heterozygous *DMD-null* mice. The human dystrophin in DYS-HAC1; *DMD-null* mice improves plasma CK activity, pathohistological characteristics, and running performance^31^. Moreover, the human dystrophin derived from DYS-HAC improves the CK activity and the survival rate seen in *DMD*-knockout pigs^32^.

In this study, we investigate the differences in the sensitivity of plasma CK activity and urine/plasma titin fragment levels in WT, DYS-HAC1, *DMD-null*, and DYS-HAC1; *DMD-null* mice .

## Methods

### Ethics declaration

This study was approved by the Animal Care and Use Committee of Tottori University (Permit Numbers: 16-Y-20, 17-Y-28, 19-Y-22, 20-Y-31, 21-Y-26, and 22-Y-36). All methods were performed in accordance with the ARRIVE guidelines and relevant regulations.

### Mice

*DMD-null*^4^ and DYS-HAC1 mice^31^ were previously generated and subjected to genotyping PCR as reported previously.

### Measurement of creatine kinase activity

The blood samples (approximate 80–100 µl) from the same individual mice at 4, 8, and 24 weeks of age or individual mice at 3-6 weeks of age were collected from orbital sinus under anesthesia using heparinized micropipettes (Drummond Scientific Company) and spun down at 1000 rcf for 10 min at 4℃. The supernatant was deposited on a DRI-Chem Slide CPK-PIII (Fujifilm) and then CK activity was measured with DRI-Chem 7000 V (Fujifilm).

### Measurement of titin fragment levels

Mice were gently restrained by gathering the skin of the neck and back to hold the mouse over a hydrophobic surface to induce urination and then the urine (10–30 µl) collected with a pipetman was stored at -30℃ until analysis^17^. Urine and plasma titin concentrations were determined using Mouse Titin N-fragment (Urine) ELISA kit-IBL (Immuno-Biological Laboratories Co. Ltd.) and Mouse Titin N-fragment (Serum) ELISA kit-IBL (Immuno-Biological Laboratories Co. Ltd.), respectively, in accordance with the manufacturer’s instructions. Urine creatinine (Cr) concentrations were measured using an assay kit (LabAssay Creatinine; Wako Pure Chemical Industries, Ltd.). Urine titin fragment levels were normalized by urine Cr and are expressed as pmol/mg Cr

### Sample preparation for histological analysis

Mouse tissues were excised and frozen in cooled isopentane using liquid nitrogen. Transverse sections were made using a cryostat (Leica CM 1950, Leica) and collected onto MAS-GP glass slides (Matsunami) for hematoxylin-eosin (H&E) staining and immunofluorescence and into a 1.5-ml tube for western blotting.

### Histological analysis

H&E staining of transverse section (10 μm) was carried out using a standard method. Photomicrographs were obtained using a microscope (BZ-X810, Keyence). To measure the area of myofibers with central nuclei and total cross-sectional area, a hybrid cell count application (Keyence) was used.

### Immunofluorescence

The immunofluorescence protocol was as previously reported^31^. In brief, sections were fixed with 4% PFA for 15 min and permeabilized with 0.25% Triton X-100 for 15 min. These sections were then incubated with blocking buffer for more than 30 min, followed by incubation with primary antibodies [dystrophin (Abcam, ab15277, 1:300) and Laminin α2 (Santa Cruz Biotechnology, sc-59854, 1:300)] at 4 °C overnight and then with secondary antibodies [Alexa 594 goat anti-rabbit IgG (Invitrogen, A32740, 1:500) and Alexa 647 goat anti-rat IgG (Invitrogen, A48265, 1:500)] with DAPI for 1 h. Fluorescence was obtained using an all-in one fluorescence microscope (BZ-X810) equipped with monochrome CCD camera, Plan Fluorite 20X LD PH lens, BZ-X filter (DAPI-V, TRITC, and Cy5), and BZ-X800 analyzer software (all from Keyence). The image processing for crop was performed by photoshop.

### Western blotting

The western blotting protocol was as previously reported^31^. In brief, 10-µm-thick slices were treated using a lysis buffer (50 mM Tris-HCl pH 7.4, 150 mM NaCl, and 0.2% Triton X-100) including protease inhibitors (Roche) on ice for 15 min and then spun down at 12,000 rcf at 4 °C for 15 min. After the protein quantification of the supernatant using BCA protein assay kit (Thermo Fisher Scientific), the protein lysate was mixed with an equal amount of 2X Laemmli sample buffer containing β-mercaptoethanol, followed by boiling at 95 °C for 5 min. The samples were resolved on 3–8% Tris-Acetate gel with Tris–Acetate SDS running buffer and transferred to a PVDF membrane. The PVDF membrane was incubated with blocking buffer for 1 h, followed by incubation with primary antibodies [dystrophin (Abcam, ab15277, 1:6000), GAPDH (Cell Signaling Technology, #5174, 1:50,000)] at 4 °C overnight, and then with secondary antibody [goat anti-rabbit IgG H&L (HRP) (Abcam, ab205718, 1:15,000)] for 1 h. Signals were detected using an ECL western blotting substrate in ImageQuant LAS 4000 mini (GE Healthcare Life Sciences). HiMark Prestained Protein Standard (Thermo Fisher Scientific) and Precision Plus Protein Dual Color Standards (Bio-Rad) were used as a protein ladder.

### Statistical analysis

Dunn’s multiple comparison test and nonparametric Spearman correlation coefficient (r) analysis were carried out using Prism 8. *P* < 0.05 was considered statistically significant. When the r was < 0.4, 0.4–0.7, and ≥ 0.7, the correlation was classified as weak, moderate, and strong, respectively.

## Results

### Different sensitivity between plasma creatine kinase activity and urine/plasma titin fragment levels

**Fig. 1:**
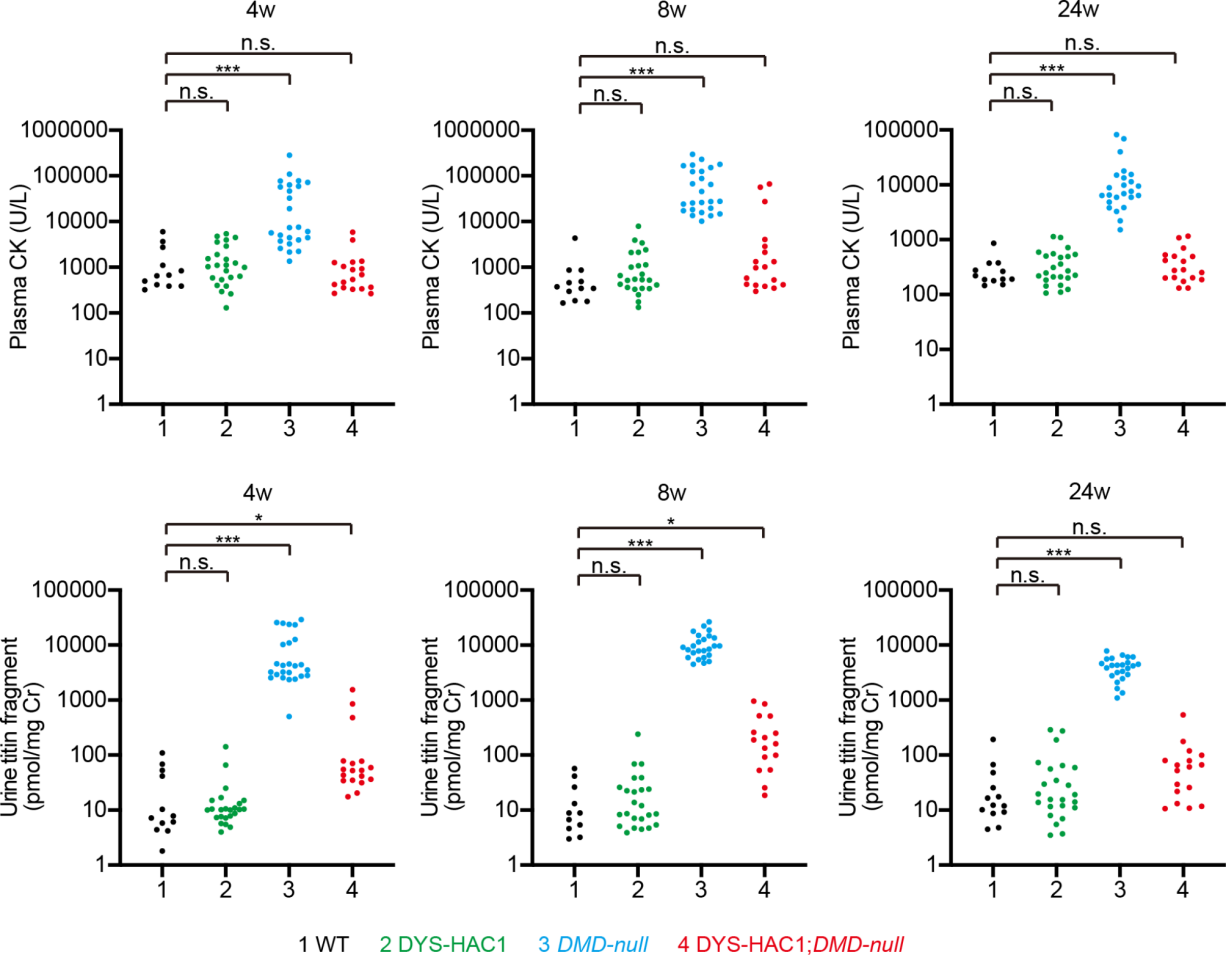
Measurement of plasma CK activity and urine titin fragment levels. The values of plasma CK activity and urine titin fragment levels in WT (*n* = 11–12), DYS-HAC1 (*n* = 24), *DMD-null* (*n* = 24–25), and DYS-HAC1; *DMD-null* mice (*n* = 16–18) at 4, 8, and 24 weeks of age. * *P* < 0.05, *** *P* < 0.001, n.s.: not significant. Dunn’s multiple comparison test was performed.

**Fig. 2:**
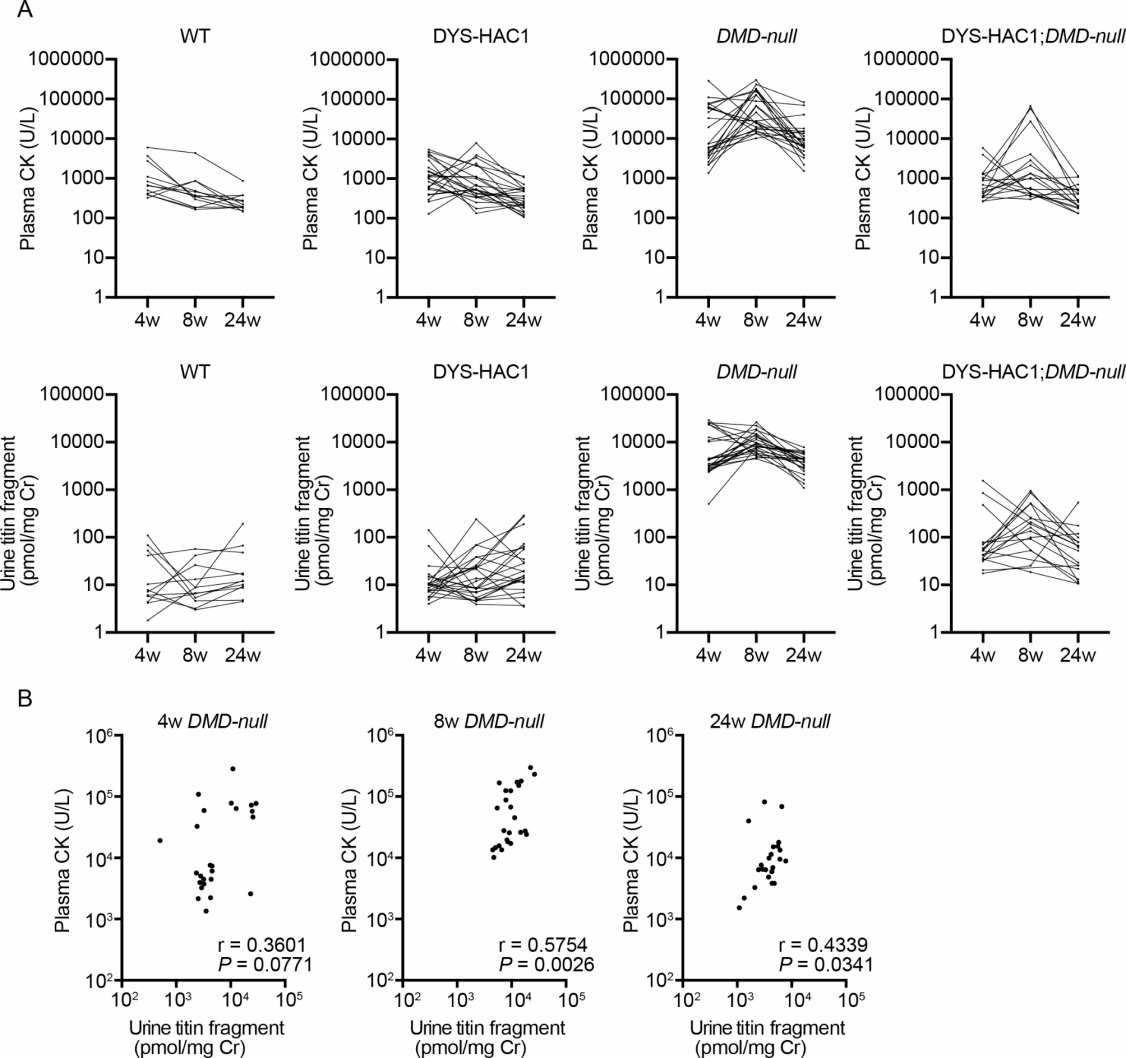
Trends and correlation of plasma CK activity and urine titin fragment levels. **A** The trends of plasma CK activity and urine titin fragment levels in WT (*n* = 11–12), DYS-HAC1 (*n* = 24), *DMD-null* (*n* = 24–25), and DYS-HAC1; *DMD-null* mice (*n* = 16–18) at 4, 8, and 24 weeks of age. **B** Nonparametric Spearman correlation coefficient analysis between plasma CK and urine titin fragment levels in *DMD-null* mice at 4, 8, and 24 weeks of age.

To compare the values of plasma CK activity and urine titin fragment levels, blood and urine were collected from wild-type (WT), DYS-HAC1, *DMD-null*, and DYS-HAC1; *DMD-null* mice at 4, 8, and 24 weeks of age (Fig. 1). Because *DMD-null* mice lack the entire 2.4 Mb dystrophin gene^4^, the values of plasma CK activity and urine titin fragment levels in *DMD-null* mice were significantly higher than those in WT mice at each timepoint (*P* < 0.001) (Fig. 1). Next, when we compared the values of plasma CK and urine titin fragment levels between *DMD-null* and DYS-HAC1; *DMD-null* mice, the value had a tendency to decrease considerably in DYS-HAC1; *DMD-null* mice (Fig. 1). Moreover, when we compared plasma CK activity between WT and DYS-HAC1; *DMD-null* mice, we found no significant difference at each timepoint. Interestingly, the value of urine titin fragment levels in DYS-HAC1; *DMD-null* mice was slightly but significantly higher than that in WT mice at 4 and 8 weeks of age (*P* < 0.05) (Fig. 1). Furthermore, there was no significant difference in the values of plasma CK activity and urine titin fragment levels between WT and DYS-HAC1 mice with the 24-week period. When we compared the values of plasma CK and urine titin fragment levels with the 24-week period, there was a trend in *DMD-null* mice to be higher at 8 weeks than at 4 and 24 weeks of age (Fig. 2A). Weak or moderate correlation between plasma CK and urine titin fragment levels in *DMD-null* mice at 4, 8, and 24 weeks of age was observed (Fig. 2B).

**Fig. 3:**
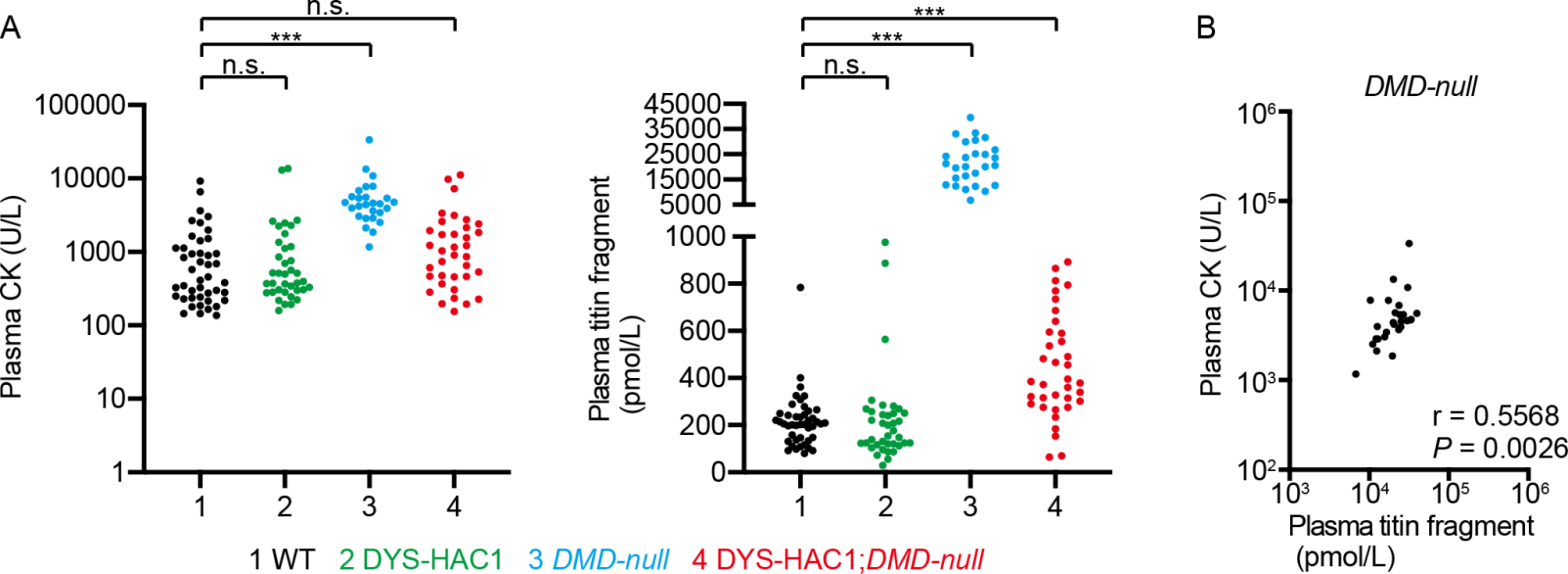
Measurement of plasma CK activity and plasma titin fragment levels. **A** The values of plasma CK activity and plasma titin fragment levels in WT (*n* = 44), DYS-HAC1 (*n* = 38), *DMD-null* (*n* = 27), and DYS-HAC1; *DMD-null* mice (*n* = 36) at 3–6 weeks of age. *** *P* < 0.001, n.s.: not significant. Dunn’s multiple comparison test was performed. **B** Nonparametric Spearman correlation coefficient analysis between plasma CK and plasma titin fragment levels in *DMD-null* mice at 3–6 weeks of age.

Similar to urine titin fragment levels, plasma titin fragment levels in *DMD-null* mice were also significantly higher than those in WT mice at 3–6 weeks of age (*P* < 0.001) (Fig. 3A). When we compared the plasma titin fragment levels between *DMD-null* and DYS-HAC1; *DMD-null* mice, the value had a tendency to decrease considerably in DYS-HAC1; *DMD-null* mice similar to the findings for urine titin fragment levels. Moreover, plasma titin fragment level in DYS-HAC1; *DMD-null* mice was also significantly higher than that in WT mice (*P* < 0.001) (Fig. 3A). Moderate correlation between plasma CK and plasma titin fragment levels in *DMD-null* mice at 3–6 weeks of age was observed (Fig. 3B).

### Histological analysis and dystrophin expression in skeletal muscles

**Fig. 4:**
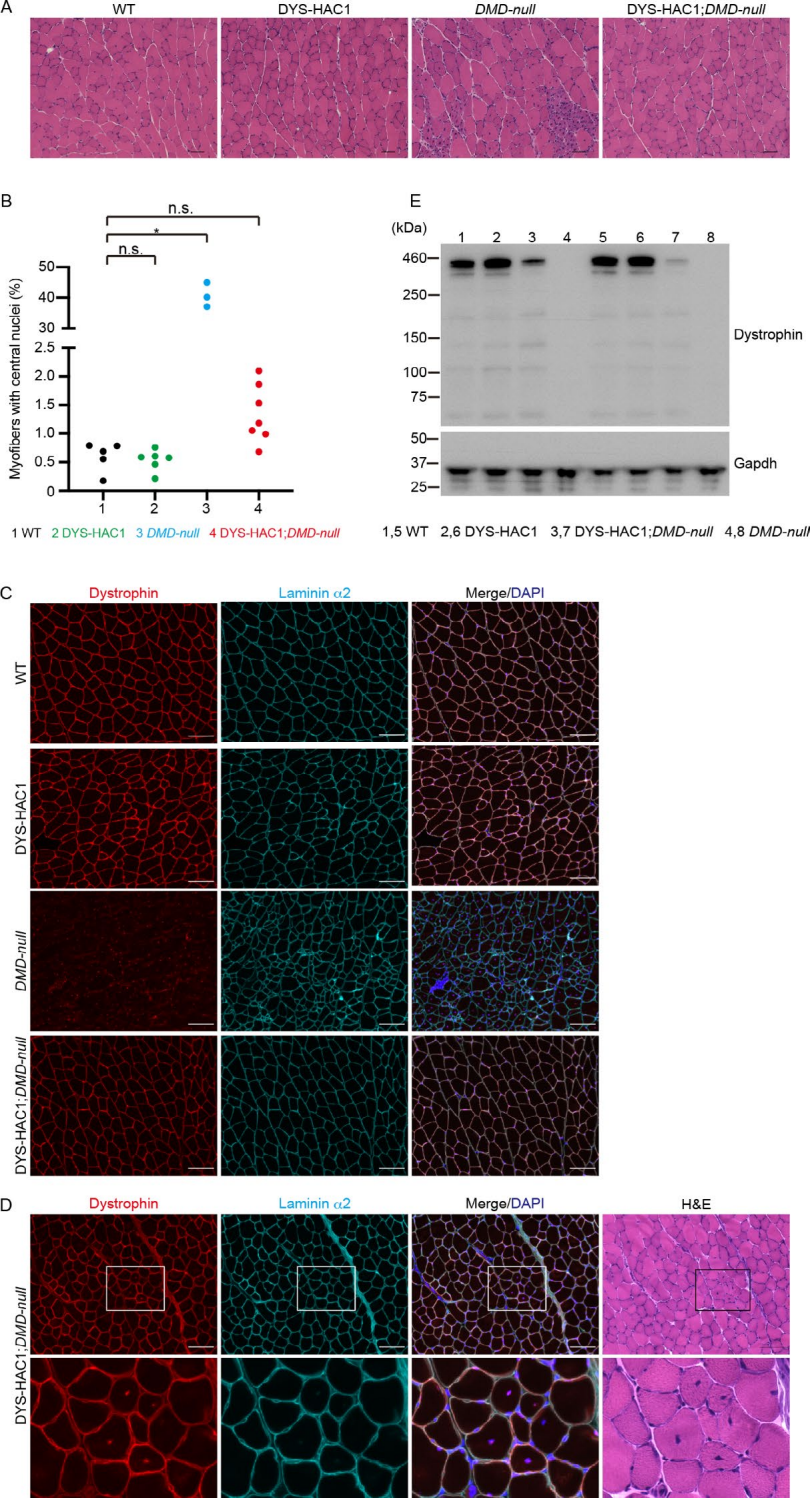
Characterization of skeletal muscles in WT, DYS-HAC1, *DMD-null*, and DYS-HAC1; *DMD-null* mice. **A** Representative images of H&E staining for gastrocnemius muscles of WT, DYS-HAC1, *DMD-null*, and DYS-HAC1; *DMD-null* mice at 7–13 weeks of age. Scale bar: 50 μm. **B** The proportion of myofibers with central nuclei in gastrocnemius muscles of WT (*n* = 5), DYS-HAC1 (*n* = 6), *DMD-null* (*n* = 3), and DYS-HAC1; *DMD-null* (*n* = 7) mice at 7–13 weeks of age. * *P* < 0.05, n.s.: not significant. Dunn’s multiple comparison test was performed. **C** Immunofluorescence for dystrophin and Laminin α2 in gastrocnemius muscles of WT, DYS-HAC1, *DMD-null*, and DYS-HAC1; *DMD-null* mice at 7–8 weeks of age. DAPI was used for staining nuclei. Scale bar: 100 μm. **D** Serial sections of H&E staining and immunofluorescence for dystrophin and Laminin α2 in gastrocnemius muscles of DYS-HAC1; *DMD-null* mice at 7 weeks of age. The square in the top panel is magnified in the bottom panel. Scale bar: 100 μm. **E** Western blotting for dystrophin in gastrocnemius muscles of WT, DYS-HAC1, *DMD-null*, and DYS-HAC1; *DMD-null* mice (each *n* = 2) at 7–8 weeks of age. Gapdh was used as a loading control.

H&E staining in WT, DYS-HAC1, *DMD-null*, and DYS-HAC1; *DMD-null* mice at 7–13 weeks of age showed that myofibers with central nuclei, a hallmark of muscle regeneration induced by muscle injury, were prominent in *DMD-null* mice, comprising approximately 40% of the total cross-sectional area (Fig. 4A and B). Meanwhile, although most of the myofibers in DYS-HAC1; *DMD-null* mice appeared to be normal and expressed human dystrophin in sarcolemma similar to WT and DYS-HAC1 mice, there was a trend for myofibers with central nuclei to be present at a slightly higher proportion in DYS-HAC1; *DMD-null* mice than in WT mice (Fig. 4A and B, and 4C). When we investigated whether the myofibers with central nuclei in DYS-HAC1; *DMD-null* mice express human dystrophin, there was no apparent difference in the localization and expression of dystrophin between the myofibers with central nuclei and peripheral nuclei in DYS-HAC1; *DMD-null* mice (Fig. 4D). To further investigate the cause of the slightly higher proportion of myofibers with central nuclei in DYS-HAC1; *DMD-null* mice, we focused on the amount of human dystrophin in skeletal muscle. Although full-length human dystrophin was expressed at approximately 426 kDa in skeletal muscles of DYS-HAC1; *DMD-null* mice, the amount of human dystrophin in DYS-HAC1; *DMD-null* mice was lower than that of mouse dystrophin in WT mice (Fig. 4E and Supplementary Figure S1). Moreover, there was variation in the amount of human dystrophin in DYS-HAC1; *DMD-null* mice (lanes 3 and 7 in Fig. 4E).

## Discussion

We compared the sensitivity of plasma CK activity and urine/plasma titin fragment levels using WT, DYS-HAC1, *DMD-null*, and DYS-HAC1; *DMD-null* mice. Similar to findings in *mdx* mice carrying a nonsense mutation in exon 23 of the dystrophin gene^16,33^, the values of plasma CK activity and urine titin fragment levels were significantly higher in *DMD-null* mice than in WT mice. The highest values of these biomarkers in *DMD-null* mice during 24-week period were found at 8 weeks. *DMD-null* mice show myofiber necrosis, infiltrations of immune cells, and muscle regeneration during crisis period at 3–4 weeks of age followed by a vigorous regenerative response^4^. Maximum CK value in DMD patients is found around 1–6 years old and the average CK activity declines according to age^34,35^. Further experiments to investigate the relationship of muscular dystrophy phenotypes to fluctuation of CK activity and titin fragment levels in long term in *DMD-null* mice would be beneficial.

Human dystrophin derived from DYS-HAC1 was localized in sarcolemma of skeletal muscle and its molecular weight was also approximately 426 kDa. Although the human dystrophin in DYS-HAC1; *DMD-null* mice was expressed at lower level than mouse dystrophin in WT mice, the phenotypes observed in *DMD-null* mice, such as the proportion of myofibers with central nuclei, plasma CK activity, and urine/plasma titin fragment levels, were improved in DYS-HAC1;*DMD-null* mice. However, the human dystrophin in DYS-HAC1;*DMD-null* mice was insufficient to completely compensate for the deficiency of mouse dystrophin on the percentage of myofibers with central nuclei in cross-section area. The lower amount of human dystrophin protein or a functional difference in dystrophin between human and mouse could be involved in this.

Considering that urine/plasma titin fragment levels but not plasma CK activity in DYS-HAC1; *DMD-null* mice were slightly higher than those in WT mice, urine/plasma titin fragment levels could be more sensitive than plasma CK activity in DMD model mice. A recent study similarly showed that urine titin fragment levels are more sensitive as a pharmacodynamic biomarker of microdystrophin efficacy compared with CK activity^21^. It would be useful to compare the sensitivity between urine and plasma titin fragment levels using WT and DYS-HAC1; *DMD-null* mice in future experiments. Considering that blood tends to be more stable than urine^36^ and glomerular filtration of plasma titin fragments could be the source of urine titin fragments, the sensitivity of titin fragment levels in plasma might be higher than that of in urine. Although Calpain, MMP-2, and MMP-12 have been involved in cleavage of titin^25,37,38^, the molecular mechanisms of how dystrophin deficiency leads to production of urine/plasma titin fragments are still unclear.

In addition to CK activity and titin fragment levels, other biomarkers have been identified in DMD. For example, protein biomarkers in urine (UMOD, NUTF2, and TNFRSF16), muscle injury biomarkers (TNNI3, CAMK2A/B, MAPK12, MDH1, and GP1), metabolites biomarkers (Creatine/creatinine ratio, Guanidinoacetic acid, and Prostaglandin D2), muscle-specific microRNAs (miR-1, miR-133a, miR-133b, and miR-206), and other microRNAs (miR-146b, miR-221, miR-155, miR-214, and miR-222) are reported^5,9,10,13–16^. To compare the difference of sensitivity between these biomarkers and titin fragment levels, DYS-HAC1; *DMD-null* mice could be beneficial.

We can take advantages of the DYS-HAC1 vector in generation of novel humanized DMD model mice carrying mutations in the human dystrophin gene using CRISPR/Cas9 genome editing system. The humanized DMD model mice would be applied not only to investigating the difference of sensitivity in some biomarkers but also to developing several therapeutic strategies using read-through mutation, exon skipping, skeletal muscle cell transplantation, and full-length/micro-dystrophin transfer^39^.

## Conclusions

Although it is difficult to compare the sensitivity using the different type of biomarkers between amount of protein and enzyme activity, urine/plasma titin fragment levels could be a more sensitive biomarker than plasma CK activity.

Supplementary Figure S1. Original blots for Fig. 4E.

Yellow dash lines indicate the clipped area in Fig. 4E. The membranes were cut prior to hybridization with antibodies.

## Electronic supplementary material

Below is the link to the electronic supplementary material.

Supplementary Material 1

## Data Availability

All data generated or analyzed during this study are included in this published article.

## Abbreviations

DMD: Duchenne muscular dystrophy
HAC: human artificial chromosome
H&E: hematoxylin-eosin
CK: creatine kinase
Cr: creatinine
WT: wild type
