# Supplementary figures and images for: Titin fragment is a sensitive biomarker in Duchenne muscular dystrophy model mice carrying full-length human dystrophin gene on human artificial chromosome
Source: Sci Rep. 2025 Jan 13;15:1778. doi: 10.1038/s41598-025-85369-5 (PMC11730604; doi:10.1038/s41598-025-85369-5)

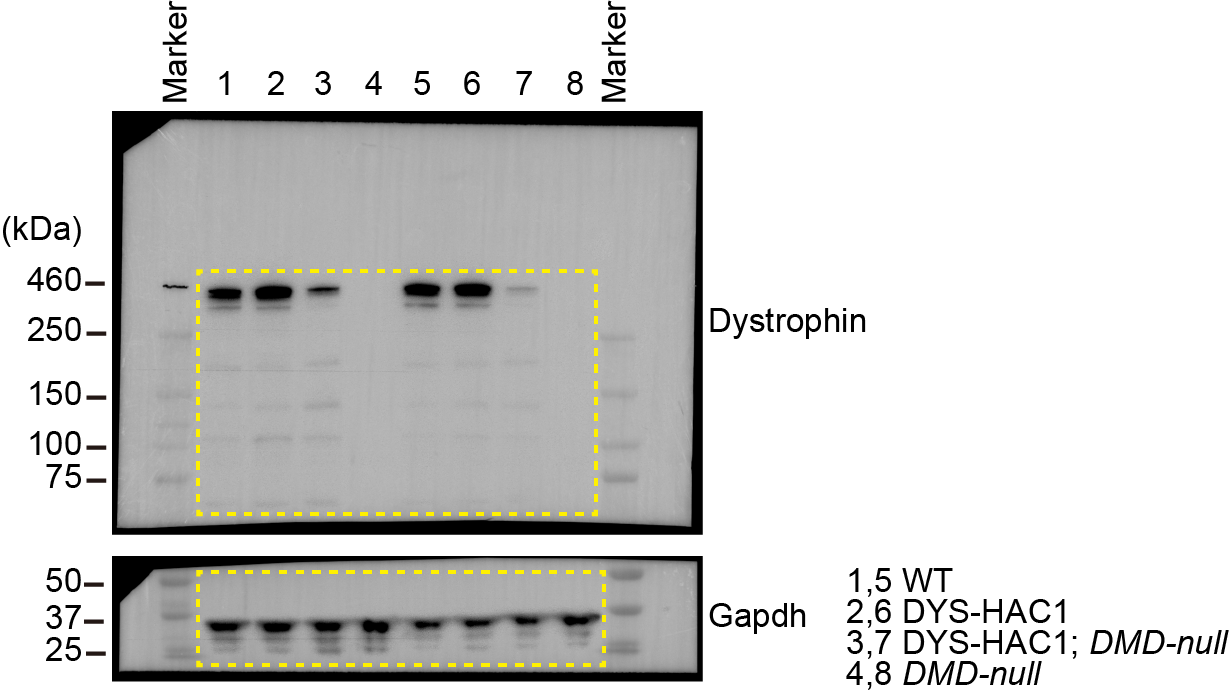

Supplement: Supplementary file 1 — Supplementary Material 1 [file 41598_2025_85369_MOESM1_ESM.tif]
